# Obtaining Microbiologically Safe Hatching Eggs from Hatcheries: Using Essential Oils for Integrated Sanitization Strategies in Hatching Eggs, Poultry Houses and Poultry

**DOI:** 10.3390/pathogens13030260

**Published:** 2024-03-18

**Authors:** Gabriel da Silva Oliveira, Concepta McManus, Igor Rafael Ribeiro Vale, Vinícius Machado dos Santos

**Affiliations:** 1 Faculty of Agronomy and Veterinary Medicine, University of Brasília, Brasília 70910-900, Brazil; gabriels.unb@gmail.com (G.d.S.O.);; 2 Laboratory of Poultry Science, Federal Institute of Brasília—Campus Planaltina, Brasília 73380-900, Brazil

**Keywords:** egg microbiology, microbial reduction, natural antimicrobials, poultry industry, poultry microbiology, poultry safety

## Abstract

Essential oils are liquids containing non-toxic compounds that are unfavorable to the growth of microorganisms. They are sold globally at affordable or very high prices, depending on the availability and type of plant, the scale of production, the extraction method, costs associated with logistics and electricity consumption, among other variables. Each year, the quantity of research dedicated to the antimicrobial potential of essential oils in poultry farming is expanding. Researchers consensually relay that this increase is due to the growing resistance of microorganisms to traditional antimicrobials and concerns about the toxicity of these products. This review proposes an analysis of the antimicrobial feasibility of using essential oils to address microbial challenges in poultry farms, aiming to ensure the production and supply of microbiologically safe hatching eggs. Based on the findings in the literature, in addition to following other necessary precautions in the daily routines of poultry farming practices, developing an antimicrobial control program with essential oils that integrates poultry facilities, poultry and hatching eggs, adapted to the particularities of each context seems to be extremely effective.

## 1. Introduction

Microbial communities are not limited to just colonizing poultry facilities, they also colonize poultry until the moment of slaughter and beyond [1]. Microbial colonization comes from the environment, maternal transmission, transmission between poultry during the consumption of feed and water, as well as human transmission [2,3,4,5,6]. Microbiological damage that compromises the health and well-being of poultry can occur much sooner than expected, resulting in widespread complications such as production cessation and significant repair expenses, requiring immediate solutions.

Poultry farms that follow quality sanitary principles make it possible to raise poultry both in confined and unconfined environments, with due restrictions on contamination of their products, including hatching eggs. Poultry feeding and watering systems, egg collection systems, ventilation and refrigeration systems, materials and equipment storage rooms and egg storage rooms, as well as parking areas, transport trucks and circulation spaces of people and vehicles, must comply with high sanitary standards. Typically, professional staff at poultry farms perform a variety of tasks to mitigate the risk of uncontrolled contamination in poultry facilities and prevent the development of avian infections [7]. Adopting appropriate work attire, following procedures using microbiologically safe materials, and strictly controlling access to facilities, are some measures adopted. However, concern about the abusive use of synthetic antimicrobials in the poultry industry, aiming to maintain high sanitary standards, has led researchers to recommend updating prevention strategies [7,8,9,10,11].

The global dissemination of essential oils as sanitizers in poultry farming [7,8,12,13,14,15] promotes the innovative “Healthy Sanitization of Poultry Farms” concept. This paradigm aims to implement effective avian health control protocols, adapted to ideal spatial conditions, with the responsibility of reducing microbial levels in the air and on contaminated surfaces and preserving the integrity of animal, human and environmental health, considering possible failures during handling and repetitive daily work. Furthermore, it is a concept that aims to encompass standards established by regulatory authorities or government bodies to ensure the supply of hatching eggs with acceptable microbiological parameters. Furthermore, the synergy between indirect antimicrobial treatment (in the poultry farming environment, as mentioned above) and direct antimicrobial treatment (in the poultry itself) [7,8,9,16] can be a way to maximize the antimicrobial benefits in poultry products. Notably, the administration of antimicrobials formulated with essential oils through diets or water to poultry provided impressive results in the production of eggs without conventional antibiotic residues and with lower microbial loads in the shell [17,18].

This review proposes an analysis of the antimicrobial feasibility of using essential oils to address microbial challenges in poultry farms, aiming to ensure the production and supply of microbiologically safe hatching eggs.

## 2. Paper Search Strategy

For this review, papers (research and review), book chapters and conference papers available on Google Scholar written in Portuguese or English up to 2 January 2024 were examined. The search terms were organized into six distinct groups, covering investigations on topics such as “microbial contamination in poultry farms”, “poultry antimicrobial management”, “essential oils”, “antimicrobial function of essential oils”, “application of essential oils in poultry farming” and “essential oils and poultry products”. The papers were thoroughly researched until each topic was comprehensively understood. Papers meeting the criteria defined for each group were chosen for inclusion, while those that did not meet the specified criteria were excluded.

## 3. Poultry Farms Are an Ideal Environment for Undesirable Microorganisms

Floors, fans, vents, feed loaders, feeders, drinkers, and wall crevices of poultry farms can be persistently contaminated with *Salmonella* spp., *Campylobacter* spp., *Escherichia coli* and/or *Staphylococcus aureus* [4]. Likewise, feed contaminated with *Salmonella* spp. and *Escherichia coli* can be fed to poultry, contributing to systemic contamination of the farm [5]. Furthermore, fungal contamination by *Aspergillus flavus*, *Aspergillus niger*, *Aspergillus fumigatus*, *Mucor* spp., *Penicillium* spp. and/or *Fusarium* spp. Can be observed in water lines, cooling pad water, fans, and floors of broiler farms [19]. These factors compromise the quality of water and air in poultry facilities. Bacterial (e.g., by *Salmonella* spp. and *Escherichi coli*) and fungal (e.g., by *Aspergillus* spp.) contamination in poultry farms harms the poultry health and the quality and viability of poultry products.

In a study carried out by Kemmett et al. [20], the bacterium *Escherichia coli* was identified in several pathological changes present in broiler chickens during the first week of life, including pericarditis, perihepatitis, abnormal liver color, ascites, cellulitis, and abnormal yolk sac. These changes are particularly concerning, as it suggests that approximately 70% of poultry mortality in the first week can be attributed to these complications [20]. Muna et al. [21] reported that young broilers contaminated with *Salmonella* spp., mainly *Salmonella enterica* subsp. *enterica* serovar Enteritidis and *Salmonella enterica* subsp. *enterica* serovar Typhimurium developed septicemia due to systemic changes and injuries in vital organs, such as the liver, intestine, spleen, heart, and brain. These changes include hepatomegaly, splenomegaly, inflammation of the intestinal mucosa, necrotic foci in the spleen, liver, and brain, as well as degeneration of the myocardial muscle fiber [21]. An outbreak of fungal infections of the respiratory tract of poultry naturally caused by *Aspergillus* spp. was reported in a poultry house [22]. These infections have resulted in significant complications, such as alveolar emphysema, atelectasis, thrombosis, and pneumonic lung with granulomatous tissue and granulomatous encephalitis [22]. These complications, in turn, contributed to the mortality of 200 approximately two-week-old broiler chickens [22].

In production systems, eggs can be horizontally contaminated by *Salmonella enterica* subsp. *enterica* serovar Typhimurium, present in poultry feces [3]. Thus, the concern arises because the eggshell is an access portal for microorganisms and is close to internal structures. In addition to *Salmonella* spp., pathogens from other genera such as *Clostridium*, *Enterococcus*, *Staphylococcus*, *Alcaligenes*, *Enterobacter*, *Escherichia*, *Klebsiella*, *Pseudomonas*, *Shigella*, *Aspergillus*, *Candida*, *Fusarium* and *Penicillium* can also lodge in the eggshell (reviewed by Oliveira et al. [23]), exposing the embryo to a more intense microbial load during critical stages of development, where the embryo’s period of vulnerability is more evident. Due to this concern, some studies have explored the severity of microbial infections during embryonic development in poultry [24,25,26]. Embryonic mortality appears to be the most common consequence [27], becoming a detriment to the productive balance of the poultry chain.

It is important to clarify that the proliferation and dissemination of high rates of microbial contamination and mortality on poultry farms is not something expected and common on farms that adopt a rigorous and correct routine in health management at all stages of the production chain.

## 4. Essential Oils and Their Bacterial and Fungal Functions

In the industrial processing of natural products, large volumes of essential oils can be extracted from aromatic plants. Conventional and green processes can extract these oils from plants, but the conventional process by steam distillation stands out among them all [28]. These essential oils, volatile liquids, have aromas similar to those of the original plant and are loaded with functional components. Studies on the chemical analysis of essential oils have revealed that oil can contain more than 20 functional compounds [29]. The heterogeneity of the chemical composition of essential oils requires chemical analysis to determine the essential oils suitable for use as an antimicrobial agent. Depending on the essential oil, the main compound may be a monoterpene, phenol, aldehyde, ketone, alcohol, hydrocarbons, or another compound (Table 1). Phenols, alcohols, and aldehydes were found to be the most effective against Gram-negative and Gram-positive bacteria, while hydrocarbons were the least effective [30]. This finding agrees with El-Baroty et al. [31], who stated that antimicrobial activity gradually decreases from phenols (with greater activity) to hydrocarbons (with lower activity).

Therefore, the chemical composition of essential oils may explain their antimicrobial functions, including effectiveness against bacteria and fungi isolated or not from poultry (Table 2). This occurs because the interaction of these compounds with the cell wall and membrane of microorganisms promotes an increase in the permeability of these structures, resulting in leakage or alteration of microbial homeostasis [39,40]. Although some essential oils have been tested effectively to combat microorganisms on poultry farms, recent studies have warned that the effectiveness of these oils depends on the dose [13,15]. *Zingiber Officinalis* essential oil reduced the bacterial growth of *Escherichia coli* (ATCC 25922) and *Staphylococcus aureus* (ATCC 11622) strains in a dose-dependent manner (400–5 µg/mL), as evidenced by Galgano et al. [41]. In agreement, Boukhatem et al. [42] reported that *Eucalyptus globulus* essential oil also inhibited, depending on the dose (20, 40 and 60 µL/disc), the growth of foodborne and/or food spoilage pathogens such as *Enterobacter sakazakii*, *Klebsiella ornithinolytica*, *Escherichia coli*, *Bacillus cereus*, *Staphylococcus aureus*, *Candida albicans*, *Candida parapsilosis*, *Saccharomyces cerevisiae*, *Trichosporon* spp. and *Aspergillus niger*. Therefore, it is essential to carefully evaluate essential oils when programming an antimicrobial formulation that meets the specific demands of each poultry farm sector. For example, the appropriate sanitizing formula for a poultry house may not be the same as that recommended for application to poultry or for sanitizing hatching eggs. Furthermore, the formulation must simultaneously act to reduce Gram-negative and Gram-positive bacteria, as well as fungi, to levels that are considered safe. Carrying out in vitro antimicrobial tests is an initial direction for developing antimicrobial formulations in poultry farming. This is because the results obtained in vitro generally reflect directly on in vivo tests [12].

## 5. Managing Poultry Farms with Essential Oils to Obtain Microbiologically Safe Hatching Eggs

### 5.1. Poultry House

Improving the relationship between poultry farms and the application of essential oils can mark substantial poultry production progress, as the antimicrobial efficacy of these oils can effectively align with management practices in poultry production sheds. An efficient sanitization plan for poultry sheds using essential oils must cover all structural and non-structural elements necessary to guarantee high-quality poultry production. Essential oils as sanitizers have proven efficiency in poultry sheds. An investigation into daily aerosol air sanitization in a poultry house during broiler farming revealed that sanitization for 60 min with a formulation containing different compounds, including 0.3% thyme, eucalyptus, and fir essential oils, in a dose of 50 mL/m^3^ of the room reduced the bacterial load in the air by 99%, without presenting toxicity to chickens [14]. In addition to broilers exposed to sanitization having a higher average body weight, than those not exposed, their blood tests indicated a significant increase in the amount of haemoglobin, lysozyme levels and bactericidal activity [14]. The nebulization of 0.5 mL of an aqueous solution of *Mentha piperita* or *Thymus vulgaris* essential oil at a concentration of 1:500 to 1:250 in poultry houses was proven effective in reducing the bacterial and fungal load in the air, drinkers, walls and/or litter [7,8]. Similarly, the combined application every three hours of *Pinus silvestris* and *Eucalyptus polybractea* essential oils at a concentration of 1:500 proved to be an efficient protocol for improving bacterial and fungal quality in the poultry air environment [16].

### 5.2. Poultry

**Figure 1:**
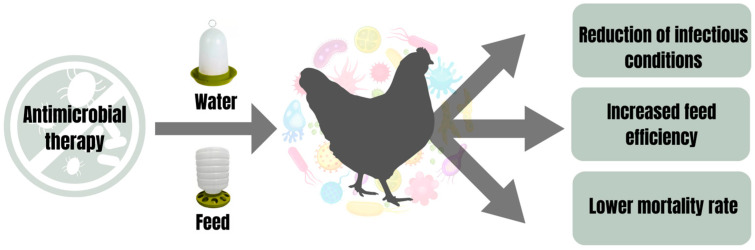
Benefits of antimicrobial therapy via water or feed in poultry.

Hatching eggs, subjected to an effective sanitization process, do not absolutely guarantee that the poultry resulting from hatching will be free of pathogens. Furthermore, even with inefficiently clean and sanitized poultry environments, this poultry can still be colonized by microorganisms present in the environment in which they live. As a result of this scenario, poultry constitutes a potential source of contamination for derived products. The main concern is centered on assessing the microbiological quality of poultry, aiming to ensure that it does not pose harmful risks to the final product or consumers. Given this need, it is recommended to subject poultry to antimicrobial therapies to guarantee both their microbiological quality and that of their final products within acceptable parameters. Studies have reported interesting results from antimicrobial treatments with essential oils via feed or water in poultry (Figure 1). Denli et al. [9] demonstrated that laying hen diets plus 150 mg/kg of *Origanum vulgare* essential oil reduced the contamination of total coliforms by 0.61 log_10_ CFU/mL and *Escherichia coli* by 1.09 log_10_ CFU/mL in eggshells. An antimicrobial treatment for layers via water-drinkers using cinnamaldehyde essential oil (diluted in a proportion of 1:8000 in drinking water) reduced the bacterial count in the cecum and eggshells [18]. Laying hens (89%) naturally infected with *Mycoplasma synoviae* (pathogen normally transmitted from breeding poultry to eggs) recovered after consuming diets supplemented with 100 mg/kg of *Melaleuca alternifolia* essential oil [17]. These authors reinforced the importance of these poultry eggs being free of conventional antibiotic residues [17]. Dietary supplementation with a blend of essential oils (containing 25% thymol and 25% carvacrol as active components, 37% silicon dioxide as caking inhibitor, and 13% glycerides as stabilizing agents; 120 mg/kg of feed) significantly reduced mortality associated with necrotic enteritis, inhibited the transport of Enterobacteriaceae in the liver and improved the intestinal integrity of broiler chickens [58]. On the other hand, diets containing 150 ppm of *Lippia origanoides* essential oil improved the feed conversion rate of layers [59].

The effects of supplying feed and water with essential oils on digestibility, feed consumption, feed conversion and, mainly, on maintaining the integrity of the intestinal health of poultry were also investigated. Barbarestani et al. [60] reported that providing feed supplemented with 600 mg of *Lavandula angustifolia* essential oil per kg of feed improved the growth performance of broilers. These improvements were mainly attributed to promoting intestinal microbiota balance, improving intestinal structure, and increasing antioxidant capacity. Abdel-Wareth and Lohakare [61] observed that the inclusion of *Mentha piperita* essential oil in the diet of laying hens at different concentrations (0, 74, 148, 222 and 296 mg/kg of feed) resulted in notable improvements in the feed intake and feed conversion ratio. Furthermore, there was a linear increase in the digestibility of crude protein, ether extract and phosphorus. These findings were directly correlated with significant improvements in the poultry laying performance. Providing drinking water for broilers enriched with 0.4 mL/L of *Lavandula angustifolia* essential oil [62] or 400 mg/L of *Satureja khuzistanica* essential oil [63] resulted in significant improvements in performance indices, including feed conversion. This improvement was also observed when laying hens received drinking water containing 0.2 to 0.3 mL/L of a mixture of essential oils from *Origanum vulgare*, *Mentha piperita* and *Pimpinella anisum* [64]. Diet supplemented with 15 mg/kg of e *Origanum vulgare* essential oil plus 2.4 g/kg of attapulgite demonstrated significant benefits on the height of ileal villi and the composition of the intestinal microbiota of broilers [65].

### 5.3. Hatching Eggs

Sanitizing eggs for hatching is also a poultry standard to ensure eggs have fewer pathogens. A bibliographical survey by Oliveira et al. [6] reported that the sanitization of hatching eggs proved viable to reduce the microbial load of the eggshell in 85–86% of protocols carried out at the experimental level. Sanitization offers an immediate reduction in the microbial load of the shell and internal contents of the eggs, lower chances of recontaminated eggs, a better hatchability rate, microbiologically safer embryos and chicks, and healthier and more viable poultry [12,13,66,67,68,69,70]. However, in some cases, sanitization did not reduce microbial contamination of the eggshell and/or caused complications such as malformations and failure to hatch [71,72,73,74]. Most of these complications require corrections in the sanitization protocol, as they may be due to poor application and the level of toxicity of the sanitizers.

Plants naturally provide many of the active ingredients for preparing sanitizers. Some of the sanitizers currently available and tested for a commercial application are based on essential oils produced by plants. In poultry farming, microbiological tests are progressively carried out to evaluate the viability of essential oils in sanitizing hatching eggs [12,15,75]. This intensifies practices that use ecological principles for antimicrobial protection in the poultry sector and de-intensifies environmental externalities caused by environmentally harmful practices. Many trees, including *Citrus aurantifolia*, *Ocimum basilicum*, and *Allium sativum*, harbor essential oils of interest to global research centers thanks to dedicated researchers who consistently share experimental results that advance the characterization of these essential oils [11,76,77,78]. Because they are (1) active against bacteria and fungi, (2) safe for humans and animals (dose-dependent), (3) sourced from readily available plants, and they have (4) positive cost–benefit ratio and (5) application versatility, essential oils need to be continually validated to redefine poultry farming, seeking to move it away from its conventional approach and cultivate an image deeply rooted in sustainability, where natural and ecologically responsible practices are the main guide.

**Figure 2:**
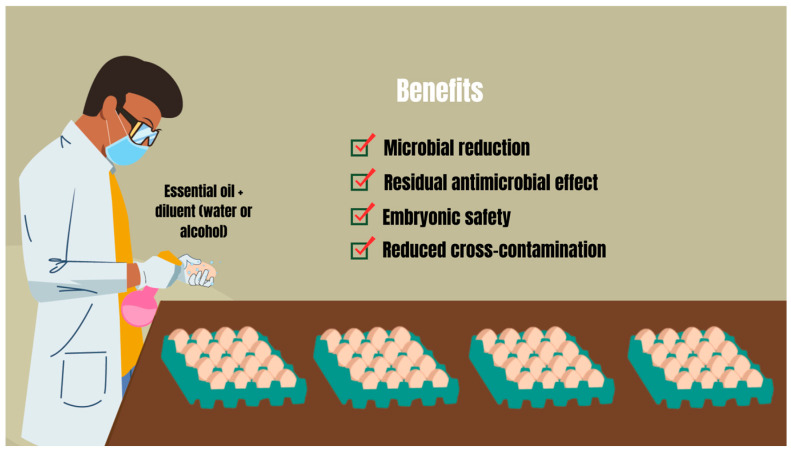
Spraying hatching eggs with essential oils and some of their benefits for poultry production. Source: Adapted from Oliveira et al. [6].

Antimicrobial therapy on poultry farms with essential oils requires a comprehensive approach. In addition to focusing on microbial control of the air, physical structure, materials, and poultry, it is necessary to integrate the sanitization of hatching eggs. This is a therapeutic complement to the cleaning activities that must be included in the management plan of poultry farms, aiming, through methods such as spraying, to reinforce the natural antimicrobial barrier of eggshells (Figure 2) [79]. This therapy may involve the use of essential oils to obtain a series of benefits that favor poultry production within appropriate microbiological quality standards (Figure 2) [15]. Mustafa et al. [80] observed that spraying *Lavandula angustifolia* essential oil significantly reduced the total count of aerobic bacteria on the eggshell surface of hatching eggs by 1.42 log_10_. Before the eggs hatched, this reduction was still significantly 0.52 log_10_ [80]. Likewise, Oliveira et al. [12] highlighted that after 1 h of spraying on hatching eggs, *Syzygium aromaticum* essential oil (0.39%) significantly reduced the total count of aerobic mesophilic bacteria and Enterobacteriaceae in eggshells by 1.19 log_10_. In addition to essential oils (1%) demonstrating the ability to reduce the bacterial load in eggshells after collection, a significant fungal reduction of 0.55 log_10_ and 0.45 log_10_ was also evidenced after immersing the eggs for 10 s in the essential oil of *Cymbopogon flexuosus* and *Lippia rotundifolia*, respectively [81].

Eggs subjected to sanitization with essential oils of *Citrus aurantifolia*, *Ocimum basilicum* and *Allium sativum* demonstrated significantly lower mean counts for total aerobic mesophilic bacteria (2.41 log_10_ CFU/mL) and Enterobacteriaceae (0.34 log_10_ CFU/mL) compared to non-sanitized eggs (5.12 ± 0.10 and 3.25 ± 0.75 log_10_ CFU/mL, respectively) (Table 3). The sanitizer based on *Allium sativum* essential oil demonstrated the greatest efficiency in reducing the bacterial load of the eggshell, resulting in a significant reduction of 3.25 log for total aerobic mesophilic bacteria and Enterobacteriaceae (Table 3). The three essential oils are comparable to formaldehyde (Table 3; unpublished data). Therefore, the essential oils used to date to sanitize eggs meet the recommendations of previous studies to balance microbiological efficiency with environmental responsibility and health preservation, as they are biodegradable, healthy, available, and efficient antimicrobial materials without serious impacts on the environment.

The hatchability rates of an incubation cycle may be associated with the toxicity and antimicrobial profile of the compounds used to sanitize hatching eggs [12,82]. Oliveira et al. [12] reported that the greatest hatchability success was observed in eggs sprayed with *Syzygium aromaticum* essential oil at 0.6 mg/mL (92.37 ± 3.25%) and paraformaldehyde (94.44 ± 4.54%), which were statistically similar. However, the grain alcohol treatment resulted in a lower hatching success (85.00 ± 2.20%) compared to the paraformaldehyde treatment while the propolis treatment resulted in an approximate 43–48% lower hatchability than the other treatments. It was demonstrated that day-old chicks from eggs sprayed with 0.39% *Syzygium aromaticum* essential oil did not exhibit morphological changes in their tissues [15]. The authors suggested that this indicates no or negligible topical toxicity of *Syzygium aromaticum* essential oil to ensure the hatching of healthy chicks [15]. Bekhet and Khalifa [82] showed that immersing eggs in a solution of 0.5% *Origanum vulgare* or *Cuminum cyminum* essential oil showed a better hatchability rate (96.21 ± 0.56% and 95.76 ± 0.94%, respectively) than eggs sanitized with alcohol (88.66 ± 1.54%), formaldehyde (82.05 ± 0.56%) and non-sanitized eggs (84.06 ± 1.54%). However, due to their oily nature, the use of essential oils in high concentrations can be disadvantageous [13], as this can result in the formation of an artificial layer that occludes the pores and potentially affects gas exchange of embryos until hatching, leading to reduced hatchability rates [83]. This argument is supported by results from several studies on table egg coatings that have proven the efficient contribution of essential oils in minimizing water and gas loss from eggs [52,53,54,55,56,84]. No negative effects were reported on the timing or hatch window of chicks from eggs sanitized with 0.39% *Syzygium aromaticum* essential oil, 0.2–0.4% *Cuminum cyminum*, or 0.2–0.4% *Origanum vulgare* [69,85].

## 6. Conclusions

In summary, the antimicrobial effects of essential oils bring significant benefits to poultry farming, contributing to the reduction of pathogen load in poultry houses and promoting positive effects on digestibility and feed consumption, improving feed conversion and the health of the poultry intestinal tract. Additionally, they help reduce the microbiota on eggshells and improve hatchability rates. It is important to highlight that essential oils are an antimicrobial treatment option that has been accepted for administration in ovo. This in ovo delivery device is a carefully researched topic in poultry farming mainly to overcome challenges of post-hatch poultry vaccination, to improve poultry production efficiency and to protect or treat poultry from pathogenic microbial infections [86,87]. Therefore, future studies also need to focus especially on the use of essential oils to prevent the growth of pathogenic bacteria in the embryonic development microenvironment and their effects on productivity. Implementing interconnected therapies using essential oils via feed, drinking water and sanitation (depending on the production stage) can be an effective strategy to combat primary and secondary contamination on poultry farms, generating synergistic effects and optimizing the results of systemic treatment. This approach needs to involve the integrated application of several therapies with essential oils, from entry into the farm until the transport of eggs to the hatcheries, contributing to maintaining an environment with safe microbiological levels throughout the process. Ensuring the availability of microbiologically safe eggs for the hatchery represents the first step to generating healthy chicks destined for farms. It is proposed to use essential oils as a microbial control agent in the poultry sector, suggesting their integrated application as follows: sanitization of poultry sheds with *Thymus vulgaris* essential oil in a proportion of 1:500–1:250 and incorporation of 150 mg/kg of *Origanum vulgare* in poultry feed. After laying, sanitize the hatching eggs with *Syzygium aromaticum* essential oil 0.39%.

## Figures and Tables

**Table 1:** Main compound of different essential oils.

Essential Oil	Main Compound	Reference
*Cymbopogon winterianus*	Citronellal (41.80%)	[32]
*Eucalyptus paniculata*	α-pinene (55.47%)	
*Lavandula angustifolia*	1,8-cineole (46.78%)	
*Origanum vulgare*	Carvacrol (69.1%)	[33]
*Thymus vulgaris*	Thymol (45.5%)	
*Lippia sidoides*	Thymol (77.2%)	
*Allium sativum*	Diallyl disulfide (40%)	[34]
*Carapa guianensis*	Limmonoids (2–5%)	
*Syzygium aromaticum*	Eugenol (92.3%)	
*Zingiber officinale*	Zingiberene (33%)	
*Citrus sinensis*	Limonene (95.48%)	
*Mentha piperita*	Menthol (55%)	
*Piper nigrum*	α-pinene (30%)	
*Citrus aurantium*	Limonene (84.3%)	[35]
*Cinnamomum camphora*	1,8-cineole (54.0%)	
*Curcuma longa*	Turmerone (31.4%)	
*Morinda citrifolia*	Octanoic acid (78.9%)	
*Plectranthus amboinicus*	Carvacrol (17.9%)	
*Petroselinum crispum*	Myristicin (40.3%)	
*Pittosporum senacia*	Myrcene (62.2%)	
*Syzygium coriaceum*	(E)-β-ocimene (24.4%)	
*Syzygium samarangense*	β-pinene (21.3%)	
*Origanum majorana*	Terpinene-4-ol (22%)	[36]
*Rosmarinus officinalis*	1,8-cineole (40%)	
*Trachyspermum ammi*	Thymol (45.17%)	[37]
*Matricaria recutita*	E-β-farnesene (34.61%)	
*Ocimum basilicum*	Estragole (87.58%)	
*Cinnamomum cassia*	E-cinnamaldehyde (82.05%)	
*Coriandrum sativum*	Linalool (43.67%)	
*Eugenia caryophyllus*	Eugenol (84.58%)	
*Cymbopogon citratus*	Citral (75.16%)	
*Lavandula stoechas*	Camphor (32.54%)	
*Origanum compactum*	Carvacrol (57.21%)	
*Cymbopogon martinii*	Geraniol (81.05%)	
*Cinnamomum zeylanicum*	Cinnamaldehyde (68.31%)	[38]
*Melaleuca alternifolia*	Terpinen-4-ol (42.65%)	
*Thymus satureioides*	Borneol (32.33%)	
*Rosemary officinalis*	1,8-cineole (49.49%)	

**Table 2:** Summary of essential oils’ antibacterial and antifungal capacity against bacteria and fungi isolated or not from poultry.

Essential Oil	MIC	SIM	SM	Reference
*Lippia origanoides*	40 μL/mL	*Escherichia coli*	Isolated	[43]
		*Staphylococcus aureus*		
*Lippia rotundifolia*	80 μL/mL	*Escherichia coli*	Isolated	[43]
	160 μL/mL	*Staphylococcus aureus*		
*Litsea cubeba*	17.72 mg/mL	*Salmonella* Typhimurium	Isolated	[44]
	8.86 mg/mL	*Yersinia enterocolitica*		
	1.11 mg/mL	*Listeria monocytogenes*		
		*Enterococcus durans*		
	17.72 mg/mL	*Enterococcus faecium*		
		*Enterococcus faecalis*		
*Origanum vulgare*	2.37 mg/mL	*Salmonella* Typhimurium	Isolated	[44]
	0.59 mg/mL	*Yersinia enterocolitica*		
	1.18 mg/mL	*Listeria monocytogenes*		
		*Enterococcus durans*		
	2.37 mg/mL	*Enterococcus faecium*		
		*Enterococcus faecalis*		
*Origanum majorana*	4.47 mg/mL	*Salmonella* Typhimurium	Isolated	[44]
		*Yersinia enterocolitica*		
	17.88 mg/mL	*Listeria monocytogenes*		
		*Enterococcus durans*		
		*Enterococcus faecium*		
*Thymus vulgaris*	2.34 mg/mL	*Salmonella* Typhimurium	Isolated	[44]
		*Yersinia enterocolitica*		
		*Listeria monocytogenes*		
		*Enterococcus durans*		
		*Enterococcus faecium*		
		*Enterococcus faecalis*		
*Cinnamomum zeylanicum*	2.52 mg/mL	*Escherichia coli*	Isolated	[45]
*Cymbopogon citratus*	1.118 mg/mL			
*Litsea cubeba*	1.106 mg/mL			
*Ocimum basilicum*	9.15 mg/mL			
*Mentha piperita*	1.14 mg/mL			
*Pelargonium graveolens*	17.8 mg/mL			
*Syzygium aromaticum*	1.318 mg/mL			
*Cymbopogon winterianus*	50–500 μL/mL	*Staphylococcus aureus*	ATCC	[46]
*Clausena heptaphylla*		*Bacillus cereus*		
*Cinnamomum tamala*		*Bacillus subtilis*		
*Ocimum sanctum*		*Salmonella* Typhimurium		
		*Escherichia coli*		
Cinnamon	0.1%	*Escherichia coli*	CECT	[47]
Clove		*Salmonella* Typhimurium		
White thyme				
*Satureja hortensis*	0.07 μL/mL	*Escherichia coli*	Isolated	[48]
	0.31 μL/mL	*Salmonella* Enteritidis		
*Syzygium aromaticum*	50–0.39%	*Escherichia coli*	ATCC	[12]
		*Pseudomonas aeruginosa*		
		*Staphylococcus aureus*		
*Citrus latifolia*	10 mL (pure)	*Bacillus cereus*	ATCC	[49]
		*Bacillus subtilis*		
		*Escherichia coli*		
		*Salmonella* Enteritidis		
		*Salmonella* Typhimurium		
		*Staphylococcus aureus*		
*Melaleuca alternifolia*	10 µL (pure)	*Salmonella* Typhimurium	ATCC	[33]
*Origanum vulgare*		*Staphylococcus aureus*		
*Pelargonium graveolens*				
*Eucaliptus globulus*				
*Cymbopogon citratus*				
*Citrus paradis*				
*Thymus vulgaris*				
*Cinnamomum cassia*				
*Citrus bergamia*				
*Cymbopogon winterianus*				
*Lippia sidoides*				
*Rosmarinus officinalis*				
*Syzygium aromaticum*				
*Mentha spicata*				
*Cinnamomum glaucescens*				
*Ocimum gratissimum*				
*Citrus limonum*				
*Citrus sinensis*				
*Citrus aurantifolia*				
*Zingiber officinale*	1%	*Escherichia coli*	ATCC	[50]
*Cymbopogon citratus*		*Staphylococcus aureus*	ATCC	
*Citrus aurantifolia*				
*Piper nigrum*	10 mL (pure)	*Bacillus cereus*	ATCC	[51]
*Petroselinum crispum*		*Bacillus subtilis*		
*Ocimum basilicum*		*Escherichia coli*		
		*Salmonella* Enteritidis		
		*Salmonella* Typhimurium		
		*Staphylococcus aureus*		
*Allium sativum*	500–100 mg/mL	*Escherichia coli*	ATCC	[52]
		*Staphylococcus aureus*		
*Ocimum basilicum*	300–100 mg/mL	*Escherichia coli*	ATCC	[53]
		*Staphylococcus aureus*		
*Citrus aurantifolia*	1%	*Escherichia coli*	ATCC	[54]
		*Staphylococcus aureus*		
*Rosmarinus officinalis*	1%	*Escherichia coli*	ATCC	[55]
		*Staphylococcus aureus*	ATCC	
*Litsea cubeba*	13.29 mg/mL	*Candida albicans*	Isolated	[44]
	1.33 mg/mL	*Candida guilliermondii*		
	13.29 mg/mL	*Candida tropicalis*		
		*Candida parapsilosis*		
	1.77 mg/mL	*Candida krusei*		
	13.29 mg/mL	*Saccharomyces cerevisiae*		
*Origanum vulgare*	1.89 mg/mL	*Candida albicans*	Isolated	[44]
	0.95 mg/mL	*Candida guilliermondii*		
	3.79 mg/mL	*Candida tropicalis*		
	1.89 mg/mL	*Candida parapsilosis*		
		*Candida krusei*		
	4.73 mg/mL	*Saccharomyces cerevisiae*		
*Origanum majorana*	13.41 mg/mL	*Candida albicans*	Isolated	[44]
		*Candida guilliermondii*		
		*Candida tropicalis*		
		*Candida parapsilosis*		
		*Candida krusei*		
		*Saccharomyces cerevisiae*		
*Thymus vulgaris*	14.05 mg/mL	*Candida albicans*	Isolated	[44]
	0.94 mg/mL	*Candida guilliermondii*		
	14.05 mg/mL	*Candida tropicalis*		
		*Candida parapsilosis*		
	1.87 mg/mL	*Candida krusei*		
	1.41 mg/mL	*Saccharomyces cerevisiae*		
*Cymbopogon winterianus*	50–500 μL/mL	*Aspergillus niger*	ATCC	[46]
*Clausena heptaphylla*		*Aspergillus fumigatus*		
*Cinnamomum tamala*		*Saccharomyces cerevisiae*		
*Ocimum sanctum*		*Candida albicans*		
*Cinnamomum cassia*	40 μL (pure)	*Candida albicans*	ATCC	[56]
*Melaleuca alternifolia*				
*Eucalyptus globulus*				
*Eugenia caryophyllus*				
Thyme	0.2%	*Aspergillus niger*	Isolated	[57]
		*Aspergillus flavus*		
		*Aspergillus fumigatus*		
		*Candida albicans*		
Anise	0.5%	*Aspergillus niger*	Isolated	[57]
		*Aspergillus flavus*		
		*Aspergillus fumigatus*		
		*Candida albicans*		
Cinnamon	0.1%	*Aspergillus niger*	Isolated	[57]
		*Aspergillus flavus*		
		*Aspergillus fumigatus*		
		*Candida albicans*		

MIC, minimum inhibitory concentration; SIM, sensitive isolated microorganism; SM, source of the microorganism; ATCC, American type of culture collection; and CECT, Spanish type of culture collection.

**Table 3:** The bacterial count of eggshells sanitized with *Citrus aurantifolia*, *Ocimum basilicum* and *Allium sativum* ^1^ essential oils *.

Treatments	Concentration Sanitizer	Application Method	Number of Eggs	TAMB (log_10_ CFU/mL)	Enterobacteriaceae (log_10_ CFU/mL)
Non-sanitized eggs	-	Spraying	12	5.12 ± 0.10 ^a^	3.25 ± 0.75 ^a^
Grain alcohol	93.8%	Spraying	12	4.51 ± 0.33 ^a^	3.00 ± 0.37 ^ab^
Formaldehyde	1.5%	Spraying	12	2.39 ± 0.49 ^bc^	1.37 ± 1.19 ^bc^
*Citrus aurantifolia*	1% ^2^	Spraying	12	2.28 ± 0.50 ^bc^	0.00 ± 0.00 ^c^
*Ocimum basilicum*	1% ^2^	Spraying	12	3.09 ± 0.23 ^b^	1.02 ± 0.89 ^c^
*Allium sativum*	1% ^2^	Spraying	12	1.87 ± 0.54 ^c^	0.00 ± 0.00 ^c^
*p* value	-	-	-	<0.0001	<0.0001

^a–c^ Different letters in the same column indicate significant differences among means (*p* < 0.05). ^1^ Bacterial counting using the eggshell washing method. ^2^ Oils at a concentration of 300 mg/mL of DMSO were used. Abbreviation: TAMB, total aerobic mesophilic bacteria. * unpublished data.

## Data Availability

Not applicable.
